# American Medical Association

**Published:** 1872-05

**Authors:** 


American Medical Association.—The minutes of the late session are now in press, and will shortly be issued in pamphlet form. Physicians can obtain them of the Secretary Wm. B. Atkinson, 1400 Pine Street, Philadelphia. Price 50 cents.

